# Willingness to use a rapid diagnostic test for malaria in a rural area of central Côte d’Ivoire

**DOI:** 10.1186/1471-2458-12-1089

**Published:** 2012-12-18

**Authors:** Colombe Coffie Comoé, Allassane F Ouattara, Giovanna Raso, Marcel Tanner, Jürg Utzinger, Benjamin G Koudou

**Affiliations:** 1 Département Environnement et Santé, Centre Suisse de Recherches Scientifiques en Côte d’Ivoire, Abidjan, Côte d’Ivoire; 2 Département de Sociologie, Université Félix Houphouët-Boigny, Abidjan, Côte d’Ivoire; 3 Laboratoire de Cytologie et de Biologie Animales, Unité de Formation et de Recherche Sciences de la Nature, Université Niangui Abrogoua, Abidjan, Côte d’Ivoire; 4 Department of Epidemiology and Public Health, Swiss Tropical and Public Health Institute, Basel, Switzerland; 5 University of Basel, Basel, Switzerland; 6 Centre for Neglected Diseases, Liverpool School of Tropical Medicine, Liverpool, UK

**Keywords:** Malaria, Rapid diagnostic test, Blood, Blood-related disease, Social representation, HIV, Côte d’Ivoire

## Abstract

**Background:**

Malaria mortality is mainly a direct consequence of inadequate and/or delayed diagnosis and case management. Some important control interventions (e.g. long-lasting insecticidal nests) have contributed to reduce malaria morbidity and mortality in different parts of the world. Moreover, the development and effective use of rapid diagnostic tests (RDTs) hold promise to further enhance the control and elimination of malaria, particularly in areas where health services are deficient. The aim of this study was to determine knowledge, attitudes, practices and beliefs in relation to RDTs for malaria in rural Côte d’Ivoire.

**Methods:**

One hundred individuals from Bozi and Yoho who sought care at the health centre in Bozi and were offered an RDT for malaria were interviewed in April 2010 using a pre-tested questionnaire on practice and perceptions in relation to RDTs for malaria. The relationships between acceptance of RDTs and factors related to opinions were identified, using generalized linear mixed models. Qualitative data from open-ended questions complemented the quantitative analysis.

**Results:**

Only 34 out of 100 patients who were offered an RDT for malaria were willing to undergo the test. People who perceived blood as a *sacred body fluid* were less likely to comply with an RDT. The concurrent availability and use of RDTs for HIV and malaria was associated with an unwilling attitude towards RDTs for malaria (Fisher’s exact test, p <0.001). The initial willingness of patients to accept malaria testing with RDTs was significantly related to general fear and wanting to know malaria infection status. For further and regular use of RDTs, a strong relationship was observed between acceptance and the idea that an RDT is a pretext used by health worker to know HIV status (odds ratio (OR) = 16.61, 95% confidence interval (CI) = 1.03-268.5). Those thinking that blood samples were useful for medical diagnoses were 8.31-times (95% CI = 2.22-31.1) more likely to undergo an RDT compared to those rejecting blood sampling as a diagnostic strategy.

**Conclusion:**

Socio-cultural factors might be barriers for accepting RDTs in general health services. There are social representations of malaria and HIV/AIDS, symbolic for blood or experiences in relation to blood taking and blood-related diseases in relation to the introduction and routine use of RDTs. Special attention should be given to these barriers as otherwise the promotion of RDTs for prompt and effective diagnosis and subsequent management of malaria is hampered.

## Background

Malaria is still an important public health problem in sub-Saharan Africa and elsewhere in the developing world [1]. Children under the age of five years and pregnant women are the most vulnerable groups [2]. Morbidity and mortality resulting from this parasitic disease remain serious obstacles for the social and economic development of the most affected regions [3,4]. Prevention and control emphasising the use of long-lasting insecticidal nets [5,6], indoor residual spraying [7] and prompt diagnosis and adequate management of uncomplicated malaria [8], have significantly reduced morbidity and mortality in many endemic areas of Africa [9-11]. However, primarily in remote rural areas, malaria remains a major public health issue due to inadequate care [12] and the absence of effective diagnosis and treatment [13]. Although symptomatic diagnosis has been successfully performed in some remote areas [14,15], in high transmission settings, it has often proven problematic [16-18] leading to an overuse of antimalarial drugs. Usually, patients who present with fever at a health facility are given artemisinin-based combination therapies (ACTs) [19,20] without prior parasitological diagnosis [21,22].

Rapid diagnostic tests (RDTs) for malaria provide an opportunity for improved point-of-care diagnosis and better disease management in malaria-endemic areas [23]. RDTs for malaria, similar to RDTs used to detect the human immunodeficiency virus (HIV), can be utilized at the point-of-care. RDTs are easy to use and provide reliable results within 15–20 minutes [24,25]. Compared to other diagnostic methods (e.g. Giemsa-stained thick and thin blood films examined under a microscope), RDTs are highly cost-effective [26-28]. Consequently, RDTs have become essential tools in malaria control and elimination [20,29-31].

Although several studies have been carried out to investigate the diagnostic performance (i.e. sensitivity, specificity and predictive values) of RDTs for malaria [32-35], including willingness to pay for such tests [28], there is a paucity of community-based studies investigating the acceptability and appropriateness of RDTs in relation to people’s beliefs and practices. The purpose of this study was to determine the knowledge, attitudes, practices and beliefs of two rural communities in central Côte d’Ivoire with regard to RDTs for malaria.

## Methods

### Ethical considerations

The study protocol was reviewed by the institutional research commission of the Centre Suisse de Recherches Scientifiques en Côte d’Ivoire (CSRS; Abidjan, Côte d’Ivoire) and received formal approval by the national ethics committee of Côte d’Ivoire. The heads of households in the study villages were informed about the objective and procedures of the study.

Oral consent was obtained from each patient (or legal guardian/caretaker for minors) before using an RDT performed on a finger-prick blood sample. Illiteracy rate is very high in the study area leading to an oral rather than written informed consent. The aims, procedures and data confidentiality were explained to the participants, so that they could make an informed decision of whether or not they wanted to be enrolled. Participation was voluntary with no further obligations for those who declined having an RDT performed. All patients were offered free treatment according to the diagnoses and the national treatment guidelines.

### Study area and population

The study was carried out in the Bozi health centre located in the district of Bouaflé in central Côte d’Ivoire (geographical coordinates: 06°55.151’ N latitude, 05°32.080’ W longitude). This health centre serves mainly two villages, Bozi itself and the neighbouring village Yoho. The two villages are situated along the Bandama River. The mean annual rainfall is approximately 1,000 mm and the average temperature is about 27°C. At the time of our study, there were 1,847 inhabitants in Bozi and 1,989 in Yoho [36]. Bozi people belong to the *Baoulé* ethnic group, whereas Yoho people are *Yowèrè*.

The health centre in Bozi had been established in 1975 by a private donor, and is currently managed by a nurse and a midwife. Two medical assistants complement the health staff. Medical consultations are provided on a daily basis and there are a few hospital beds available for in-patients. Moreover, the Bozi health centre is used for vaccinations of newborns and antenatal consultations.

For many years, patients visiting the Bozi health centre received free treatment, including antimalarial drugs, facilitated by regular provision of subsidized supplies by the external grantee. The only financial contribution requested by those who sought health care pertained to drugs prescribed by the medical staff that were not available in the pharmacy of the Bozi health centre. From 1994 onwards, a payment scheme was established for any services received by patients visiting Bozi health centre due to the termination of external funds. This change of policy from free treatment to a paying scheme resulted in a substantial reduction of health service utilisation. At the time of the current study, there were no other health centres within a radius of 10 km from Bozi.

### Data collection

In February 2010, a total of 100 malaria RDTs (ICT ML01 Malaria *Pf* test; ICT Diagnostics, Cape Town, South Africa) were made available to the Bozi health centre by the research team. Medical staff was trained to use and report results of malaria RDTs. Health workers offered to perform an RDT for malaria free of charge to those patients who presented with fever (i.e. axillary temperature >37.5°C). Using lay terms, patients were explained the opportunity to have a finger-prick blood sample subjected to an RDT for malaria in order to prescribe appropriate medication. Patients without fever and those living outside Bozi and Yoho were not eligible for enrolment.

Within two months, a total of 100 people who met our inclusion criteria were offered an RDT for malaria, but not all of them were willing to have an RDT performed. In April 2010, one of the authors (CCC), accompanied by a key informant from Bozi, conducted a cross-sectional survey. The objective was to identify and visit all the 100 people in their homes and to interview them with a pre-tested questionnaire. The questionnaire addressed five main themes: (i) characterization of the person who was offered an RDT (e.g. demographic features, educational attainment, religion, etc.); (ii) knowledge, attitudes and practices of malaria therapy; (iii) perception of blood and blood-related diseases; (iv) perception of RDTs for malaria, including reasons for acceptance or refusal to have the test performed; and (v) socio-cultural ideologies related to RDTs.

Additionally, health care providers (nurse and midwife), traditional healers and religious leaders were interviewed to gather specific information on patients’ acceptance or rejections of RDTs. Interviews with medical staff aimed at identifying barriers of using RDTs, which were further investigated in a series of in-depth interviews with the respective patients. Questions addressed to a traditional healer were focused on patients’ treatment and the importance of taking a blood sample for the diagnosis prior to prescribing an antimalarial drug. Religious leaders were interviewed for their opinions of their congregations about treatment and acceptance of modern health care provided at health care facilities. Particular emphasis was placed on the rules and arguments forwarded by religious leaders that forbid the use of blood for medical examination. The questionnaire and topic guides (in French) are available from the authors upon request.

### Data analysis

Analyses were performed using R software version 2.10.1 (the R Foundation for Statistical Computing, 2009). Generalized linear mixed models were utilized to calculate odds ratios (ORs). Response variables were the acceptance of malaria RDTs that was offered free of charge during initial health seeking (first use) and acceptance to have an RDT performed once again if deemed necessary in the future (further use). Data were categorical (yes or no). We developed two separate models: (i) for “first use” and (ii) “further use”. The models used fixed effects that were selected among parameters identified through our questionnaire survey, using a backward elimination approach, removing variables with a P value <0.2, one at a time. Village was included in the models as random effect. To compare groups, χ^2^ test or Fisher’s exact test, as appropriate, were employed. ORs were used to express statistical difference, including 95% confidence intervals (CIs).

Qualitative data were gathered along the two main themes “belief” and “medical examination” in connection with RDTs for malaria. Each theme was addressed according to specific topics (e.g. blood, malaria, effectiveness and usefulness). Once data were categorized and coded in an Excel spreadsheet, the major trends and patterns were identified. More detailed descriptions, as articulated by the respondents (freely translated from French to English), were extracted to underscore and/or complement the quantitative results.

## Results

### Determinants for accepting or rejecting RDTs for malaria

From the 100 people interviewed, only 34 were willing to have an RDT done for malaria. As shown in Table 1, the proportion of people who were unwilling to accept an RDT for malaria was very high in Bozi (78.6%), but considerably lower in Yoho (36.7%) (χ^2^ = 14.62, p <0.001). The proportion of males who were willing to undergo an RDT was higher than that of females (39.3% *vs.* 25.6%). Patients in the age range of ≥42 years showed a higher willingness to undergo an RDT for malaria than their younger counterparts (45.4% *vs.* 30.0-33.3%). Married people (75.0%) were more likely to accept an RDT for malaria than singles (25.6%). Households with 1–3 children were more in favour of an RDT for malaria than smaller or larger households (exception: very large households with 7-9 children). Different religious groups showed important differences with regard to the acceptance of having an RDT performed. While a relatively high proportion of animists and Muslims were favourable to an RDT for malaria (42.1% and 40.4%, respectively), a considerably lower percentage was found for Christians (15.0%). Our population samples from the two villages showed no statistical difference with regard to sex, age, marital status, number of children and religious beliefs. However, willingness to have an RDT for malaria performed was positively correlated with the level of education.

**Table 1:** Characteristics of the study population, stratified by whether or not people were willing to have an RDT for malaria performed

**Characteristics**	**Number of people interviewed (%)**			**χ**^**2**^	**p**
	**Total**	**Willing to have an RDT for malaria performed (n=34)**	**Unwilling to have an RDT for malaria performed (n=66)**		
Village					
Bozi	70 (70.0)	15 (21.4)	55 (78.6)		
Yoho	30 (30.0)	19 (63.3)	11 (36.7)	14.62	<0.001
Sex					
Male	61 (61.0)	24 (39.3)	37 (60.7)		
Female	39 (39.0)	10 (25.6)	29 (74.4)	1.43	0.232
Age group (years)					
14-24	20 (20.0)	6 (30.0)	14 (70.0)		
25-41	69 (69.0)	23 (33.3)	46 (66.7)		
≥42	11 (11.0)	5 (45.4)	6 (54.5)	0.80	0.670
Marital status					
Single	39 (39.8)	10 (25.6)	29 (74.4)		
Married	4 (4.0)	3 (75.0)	1 (25.0)		
Free union	57 (58.0)	21 (36.8)	36 (63.2)	†	0.088
Number of children					
0	27 (27.0)	8 (29.6)	19 (70.4)		
1-3	47 (47.0)	19 (40.4)	28 (59.6)		
4-6	21 (21.0)	5 (23.8)	16 (76.2)		
7-9	3 (3.0)	2 (66.7)	1 (33.3)		
≥10	2 (2.0)	0 (0)	2 (100)	†	0.370
Educational level					
Illiterate	42 (42.0)	12 (28.6)	30 (71.4)		
Primary	40 (40.0)	13 (32.5)	27 (67.5)		
Secondary	14 (14.0)	5 (35.7)	9 (64.3)		
Higher education	4 (4.0)	4 (100)	0 (0)	†	0.050
Religious belief					
Muslim	52 (52.0)	21 (40.4)	31 (59.6)		
Christian	20 (20.0)	3 (15.0)	17 (85.0)		
Animist	19 (19.0)	8 (42.1)	11 (57.9)		
No religion	9 (9.0)	2 (22.2)	7 (77.8)	†	0.154

† Fisher’s exact test.

### Local perception of blood and blood-related diseases

As shown in Table 2, 54% of people perceived blood as a *sacred body fluid* and the majority of them were unwilling to have an RDT for malaria performed (55.6%) (χ^2^ = 4.74, p = 0.029). More than half of the people who think that *blood keeps life in the body* (55.6%) were in favour of an RDT for malaria. Most of the respondents who were unwilling to have an RDT for malaria done (83.3%) stated that malaria is not in their blood, compared to 16.7% of those who were in favour of an RDT (Fisher’s exact test, p = 0.002). The concurrent availability and use of RDTs for HIV and malaria at the same health facility was associated with an unwilling attitude towards RDTs for malaria (88.2%) (Fisher’s exact test, p <0.001). For patients opposed to an RDT for malaria, their willingness to provide a blood sample in case of illness depends on the disease (76.3%). Most of the patients who sought care at the health facility, and who were presumptively diagnosed for malaria, were willing to have an RDT for malaria done (84.8%).

**Table 2:** Percentage of local perception of blood and blood-related diseases, stratified by people’s willingness or unwillingness of using an RDT for malaria

**Local perception of blood**	**n (%)**			**χ**^**2**^	**p**
	**Total**	**Willing (n=34)**	**Unwilling (n=66)**		
Blood is *sacred body fluid*					
Yes	54 (54.0)	24 (44.4)	30 (55.6)		
No	46 (46.0)	10 (21.7)	36 (78.3)	4.74	0.029
Blood *keeps life in the body*					
No	91 (91.0)	29 (31.9)	62 (68.1)		
Yes	9 (9.0)	5 (55.6)	4 (44.4)	†	0.267
Malaria *is inside the blood*					
Yes	44 (44.0)	23 (52.3)	21 (47.7)		
No	42 (42.0)	7 (16.7)	35 (83.3)		
Don’t know	14 (14.0)	4 (28.6)	10 (71.4)	†	0.002
Know about malaria					
Don’t know	51 (51.0)	15 (29.4)	36 (70.6)		
Symptoms	38 (38.0)	10 (26.3)	28 (73.7)		
Malaria test	11 (11.0)	9 (81.8)	2 (18.2)	†	0.002
Custom allows blood test					
Yes	86 (86.0)	29 (33.7)	57 (66.3)		
No	11 (11.0)	4 (36.4)	7 (63.6)		
Don’t know	3 (3.0)	1 (33.3)	2 (66.7)	†	1.000
Religion allows blood test					
Yes	77 (77.0)	26 (33.8)	51 (66.2)		
Don’t know	13 (13.0)	4 (30.8)	9 (69.2)		
No	10 (10.0)	4 (40.0)	6 (60.0)	†	0.873
Reason to have a blood examination performed or not					
Care	50 (50.0)	22 (44.0)	28 (56.0)		
HIV test	34 (34.0)	4 (11.8)	30 (88.2)		
Fate of blood after test	8 (8.0)	2 (25.0)	6 (75.0)		
No problem	7 (7.0)	6 (85.7)	1 (14.3)	†	<0.001
Need to do blood test for a patient					
Depends on disease	59 (59.0)	14 (23.7)	45 (76.3)		
Yes	19 (19.0)	16 (84.2)	3 (15.8)		
No	13 (13.0)	3 (23.1)	10 (76.9)		
Don’t know	8 (8.0)	1 (12.5)	7 (87.5)	†	<0.001
Need to do blood test for malaria patient					
No	65 (65.0)	6 (9.2)	59 (90.8)		
Yes	33 (33.0)	28 (84.8)	5 (15.2)		
Don’t know	2 (2.0)	0 (0)	2 (100)	†	<0.001
Justification to do blood testing by suspected malaria patients					
Know malaria	65 (65.0)	6 (9.2)	59 (90.8)		
Don’t know	34 (34.0)	28 (86.4)	6 (13.6)		
Treated without test	1 (1.0)	0 (0)	1 (100)	†	<0.002

† Fisher’s exact test.

In general two distinct types of malaria were mentioned. First, *palu garçon* (“male malaria”). In the local Baoulé language the term for this type of malaria is *djè kouadjo yafua* or *palu rouge* (“red malaria”, with reference to jaundice), and the local Yowèrè language uses the term *djé kouadjo clonmon*. Both expressions are associated with the harshness related to complicated and more severe manifestations of malaria. Second, *palu femme* (“female malaria”) or *palu blanc* (“white malaria”) refers to uncomplicated milder forms of malaria. Local terms for these types of malaria are *djè kouadjo* (Baoulé) and *djé kouadio limon* (Yowèrè).

These results must be seen against the background of the common local concept that considers modern medicine as treating only the body, whereas traditional medicine addresses occult origins of a disease without resorting to the blood. As expressed by a traditional healer: “*when patients agree, I consult the spirits to enlighten me on the origin of evil before advising the plants to be used to treat the disease. I don’t use the blood of patients for their care even when it is palu garçon*”*.*

### Local beliefs of RDTs for malaria

Table 3 summarises local perceptions on RDTs for malaria. Fear was the main reason (92.3%) to avoid repeating an RDT (Fisher’s exact test, p <0.001). This fear was linked to the needle and the pain it causes when penetrating the human skin (85.7%) and the RDT test result (60.0%) (Fisher’s exact test, p = 0.023). In addition, 87.5% of patients who declined to have an RDT done for malaria did not know their HIV status, while 12.5% who accepted an RDT had already been tested for HIV or were not afraid of knowing their HIV status.

**Table 3:** Response percentage of local beliefs of RDTs for malaria, stratified by RDTs acceptance

**RDTs representation**	**n (%)**			**χ**^**2**^	**p**
	**Total**	**Willing (n=34)**	**Unwilling (n=66)**		
Opinions on RDTs					
Useful	42 (42.0)	8 (19.0)	34 (81.0)		
Dangerous	25 (25.0)	2 (8.0)	23 (92.0)		
Not useful	22 (22.0)	19 (86.4)	3 (13.6)		
Good	6 (6.0)	5 (83.3)	1 (16.7)		
Not good	5 (5.0)	0 (0)	5 (100)	†	<0.001
RDT utility					
No	67 (67.0)	10 (14.9)	57 (85.1)		
Yes	33 (33.0)	24 (72.7)	9 (27.3)	30.39	<0.001
Reason to have a blood test made					
Don’t know	39 (39.0)	2 (5.1)	37 (94.9)		
Feeling unwell	36 (36.0)	8 (22.2)	28 (77.8)		
Free of charge	25 (25.0)	24 (96.0)	1 (4.0)	†	<0.001
Fear of malaria test					
Test results	45 (45.0)	18 (40.0)	27 (60.0)		
Needle	28 (28.0)	4 (14.3)	24 (85.7)		
No fear	27 (27.0)	13 (48.1)	14 (51.9)	†	0.023
Reason for whether or not to repeat a malaria test					
Fear	39 (39.0)	3 (7.7)	36 (92.3)		
Accuracy of test	19 (19.0)	4 (21.1)	15 (78.9)		
Painful	17 (17.0)	9 (52.9)	8 (47.1)		
Don't know	17 (17.0)	17 (100)	0 (0)		
HIV status	8 (8.0)	1 (12.5)	7 (87.5)	†	<0.001
Knowledge about HIV					
Yes	98 (98.0)	33 (33.7)	65 (66.3)		
No	2 (2.0)	1 (50.0)	1 (50.0)	†	1.000
Reason for whether or not to do an HIV test					
Fear	63 (63.0)	13 (20.6)	50 (79.4)		
Curiosity	37 (37.0)	21 (56.8)	16 (43.2)	†	<0.001
HIV test rather than malaria test					
Yes	67 (67.0)	19 (28.4)	48 (71.6)		
Don't know	27 (27.0)	12 (44.4)	15 (55.6)		
No	6 (6.0)	3 (50.0)	3 (50.0)	†	0.250
Blood is used for something else					
Don’t know	35 (35.0)	11 (31.4)	24 (68.6)		
Yes	43 (43.0)	13 (30.2)	30 (69.8)		
No	12 (12.0)	5 (41.7)	7 (58.3)		
Witchcraft	10 (10.0)	5 (50.0)	5 (50.0)	1.83	0.608

† Fisher’s exact test.

Traditional treatments are in line with people’s customs and beliefs, as underscored by the following quote: “*Hospital cannot cure palu garçon (jaundice). If you have malaria and use a serum you die. Because we don’t know if malaria is a male or a female, thus giving first preference in consulting traditional doctors*”. Many of the respondents found RDTs for malaria of no use. Indeed, they expressed their opinions by saying: “*We know this disease very well, we have been several times victim of it. We do not need to be tested*” (male respondent, 40-year-old). Or, as expressed by another study participant in the unwilling group: “*People must have malaria and hemorrhoids in their body at any time. We are routinely exposed to the sun so when we are sick, we know that it is malaria. We do not need your malaria test*” (male, aged 45 years).

However, in spite of these local beliefs, some respondents were aware of the non-specificity of some malaria symptoms, as revealed by the following argument: “*When you have stomach ulcers, you feel as if you have early malaria. If this test helps to pay cheaper drugs because we do not have malaria, it is a good deal*” (35-year-old female respondent).

### Acceptance of having an RDT done for malaria

The willingness of patients to accept an RDT for malaria was significantly related to felt fears (first use: OR = 0.001, 95% CI = 0–0.12; further use: OR = 0.007, 95% CI = 0–0.40) and the desire to know if they really had malaria (first use: OR = 0.004, 95% CI = 0–0.22; further use: OR = 0.005, 95% CI = 0–0.30) (Table 4).

**Table 4:** Generalized linear mixed model results (outcome: RDT acceptance; fixed effects: parameters; random effects: village)

	**Accept malaria diagnosis with an RDT (first use)**	**Accept malaria diagnosis with an RDT (further use)**
**Parameters**	**OR (95% CI)**	**OR (95% CI)**
Blood is *sacred body fluid*	0.47 (0.01-17.5)	0.42 (0.12-1.52)
Blood *keeps life in the body*	3.61 (0.15-86.3)	7.16 (0.48-107.7)
Malaria *is inside the blood*		
Yes	0.89 (0.07-11.0)	2.55 (0.63-10.3)
Don’t know	0.53 (0.01-23.1)	1.07 (0.18-6.40)
Blood sample for medical test	5.30 (0.37-75.3)	8.31 (2.22-31.1)*
Utility of an RDT	3.64 (0.28-47.5)	0.18 (0.03-1.13)
Reason to have (or not) a malaria test done		
Free of charge	1.00	1.00
Fear	0.001 (0–0.12)	0.007 (0–0.40)*
Know HIV status	0.004 (0–0.22)	0.005 (0–0.30)*
Knowledge about HIV		
HIV test knowledge	6.28 (0.09-432.9)	1.70 (0.30-9.51)
HIV testing	0.31 (0.02-5.32)	0.61 (0.15-2.59)
Reason to have (or not) an HIV test done		
Fear	1.00	1.00
Curiosity	7.79 (0.11-534.6)	20.72 (0.55-781.1)
Difference between malaria and HIV test	0.22 (0–10.2)	0.16 (0.02-1.09)
HIV test rather than malaria test		
Yes	0.48 (0–51.9)	16.61 (1.03-268.5)*
Don’t know	0.89 (0.01-90.9)	1.88 (0.13-26.4)

CI: confidence interval, OR: odds ratio, *statistically significant difference (95% CI does not include OR of 1.00).

The acceptance of having an RDTs for malaria performed at the first time, was not associated with patients thinking that RDTs are a pretext used by health worker to know HIV status (OR = 0.48, 95% CI = 0–51.9) as well as those thinking that blood samples were necessary for medical diagnoses and other illness-related issues (OR = 5.30, 95% CI = 0.37-75.3). However, if asked whether they were willing to undertake further RDTs in future, a strong relationship was observed between acceptance and the idea that RDTs is a pretext used by health worker to know HIV status (OR = 16.61, 95% CI = 1.03-268.5). Those thinking that obtaining a blood sample was useful to diagnose for specific diseases through medical examination had an odds of 8.31 (95% CI: 2.22-31.1) to be ready to undergo an RDT for malaria in future compared to their counterparts with an opposed thinking.

## Discussion

ACTs have become the first-line treatment and the large-scale use is assured by the support of the Global Fund to Fight AIDS, Tuberculosis and Malaria (Global Fund in short), which has led to a much higher use in most endemic areas over the past decade despite the relatively high costs of these drugs. Inaccurate and/or delayed diagnosis led to an overconsumption of antimalarial that can also contribute to the development and spread of drug resistance [37]. The introduction of RDTs significantly reduces these risks [38] and provides a unique opportunity to render the approach of prompt diagnosis and treatment at peripheral level, i.e. the point-of-care, more effective [38]. Clearly, the use of RDT has opened up new ways for adequate case management. However, acceptance of RDTs still remains an issue for many endemic areas, as rural populations do not necessarily agree having their blood tested even for RDTs for malaria. Currently, little is known about social, cultural and religious factors governing people’s attitudes and behaviour that govern acceptance of RDTs.

Our study, undertaken in a rural part of central Côte d’Ivoire, revealed specific local concepts with regard to the perception of blood, blood-related diseases and having blood tested with an RDT. Interestingly, the majority of people from Bozi were unwilling to have an RDT for malaria done that was provided free of charge, but this was somewhat influenced by educational attainment. Indeed, unlike Bozi, the majority of household heads in Yoho had attended at least primary school. Moreover, the percentage of children from mothers with at least primary education was higher in Yoho [36]. According to our questionnaire survey, people believe that they know the manifestations of clinical symptoms due to malaria, and hence they do not see the necessity for an RDT prior to start with treatment. Patients from Bozi and Yoho were aware of some of the dangers posed by malaria. However, based on direct observation by our research team, residents from Bozi largely negated help-seeking as they perceived the quality of care as low. Since Yoho inhabitants paid for transportation to get to the Bozi health centre, it seems conceivable that they seek care for only serious conditions. In contrast, free testing with an RDT for malaria and access to drugs increased their willingness to do the exams. In addition, the user-provider interactions are crucial as also observed in a study in Ghana investigating the relationship between health workers and patients [39]. Malaria can be easily treated when promptly diagnosed but owing to people’s perceptions and beliefs, several barriers still exist as indicated by this and many other studies. Two key determinants are, first, inappropriate self-medication with medicinal plants or inappropriate medicine [40] and, second counterfeit or substandard medicines as revealed in studies from Nigeria [41-43].

Therapies from health centres and traditional healers were used together, showing an interest to modern medicine mixed with more traditional remedies. For the group of people who were unwilling to have malaria RDTs done, it was their belief that, traditional healers provided good and clear diagnosis of the disease. Therefore, visiting a health centre would mean to have modern drugs that will speed up their recovery.

There were various opinions from our surveys which contributed to a deeper understanding of the attitudes and behaviours of Yoho and Bozi populations with regard to RDTs for malaria. The introduction of RDTs was clearly affected by the different levels of acceptability owing to the innovative nature of this diagnostic tool and its use at different levels of the health care delivery systems. People linked their reluctance to previous experiences with blood taking and use. For instance, blood would also be used for other occult practices such as bewitching and fetishisms, among others. For people, blood was considered a sacred biofluid and the source of life. It cannot be used anyhow except when asked purposely for medical testing. This is the reason why, some people in the study area were compliant to accept RDTs for malaria, but not for other diseases. By contrast there was also another group of people who believed that for a disease like malaria, there is no need to do a diagnostic test because the signs were sufficient to confirm the idea that the disease (especially malaria) “*is not in the blood*”. Traditional representations did influence the acceptance of RDTs for malaria. One of these representations was the feeling to have the best treatment against malaria. According to their opinion, the best treatment against malaria did not require the use of blood unlike malaria RDTs. Thus, the local concepts of malaria as a disease do have a considerable influence on malaria management [44,45].

Acceptability of RDTs was also governed by the level of perceived fear. Interviewed persons expressed their fear based on the pain caused by the needle prick, but also other fears, particularly that blood samples would be utilized for checking their HIV status instead of malaria. Indeed, we found a high rate of people who refused to have an RDT performed for malaria, which might be explained by the fact that before introducing malaria RDTs at the health centre of Bozi, RDTs for HIV were already available. Patients might have been afraid to have their HIV status revealed, and hence explaining their reluctance to undergo an RDT for malaria. Reports from Tanzania showed that people had the same feeling of fear towards RDTs. Nevertheless the usage of RDTs was of little matter when they understood that this test confirmed malaria presence and helped to make effective and right prescription for the most appropriate drugs leading to cure [46]. Our findings thus emphasize the importance of information, education and communication (IEC) readily adapted to the local context.

Some social parameters had no influence on RDTs acceptance, but played a more subtle role. We noted that higher education level was a factor of better adherence to RDTs. In these rural areas those who were educated seek information from the specialized people as indicated. Thus, they received appropriate responses to their concerns and questions. Attitudes and behaviour towards RDTs was also partly motivated by the strict observance of religious prohibitions. These aspects prevented proper social integration of malaria RDTs to these rural communities.

In other areas, RDTs for malaria were well accepted by volunteers from the community. This was the most appropriate way to diagnose asymptomatic cases of malaria [47]. Urban populations were more ready and able to purchase malaria RDTs, while rural population and the poorest of the poor preferred RDTs free of charge [28]. In some countries, health workers use RDTs but adherence to the result is low [48-50]. For example, low adherence to RDTs was observed in rural parts of Burkina Faso because the health workers were more confident to the usual malaria symptoms from their experience than any new malaria test, which sometimes challenged their classical diagnosis [51] or it could be related to their inability to better perform and interpret results from the test [39].

Our study has some limitations. First, we pursued a convenience sampling and the size of our sample is relatively small (i.e. 100 RDTs for malaria were provided free of charge to a single health centre and a total of 100 people were interviewed). Second, within two months 100 individuals seeking care at the Bozi health centre met our inclusion criteria (i.e. axillary temperature >37.5°C), and hence were offered an RDT for malaria, but only about a third were willing to perform the rapid test. Due to time and budget constraints, we were unable to run the study until all 100 RDTs had been utilized. Third, our cross-sectional questionnaire survey was carried out two months after the introduction of RDTs for malaria, which might have introduced some recall bias. Finally, based on a single health centre and some observations that patients were no entirely satisfied with the overall quality of the service, it is difficult to generalize our findings. Clearly, our study was designed as an exploratory piece, and hence larger-scale studies should be undertaken to assess the full validity of the findings reported here.

## Conclusions

The present work addressed some of the social and cultural dimensions of introducing RDTs for malaria, which is essential for prompt diagnosis and adequate treatment. Acceptability is governed by socio-cultural and political factors, which need to be taken into account when tailoring integrated malaria control or elimination to a specific setting. The parallel use of RDTs for HIV and malaria calls for specific action and messages in the programmes to sensitize communities so that the two crucial point-of-care tests remain effective tools. Major challenges in the field (e.g. locally adapted IEC strategies) are ahead of us if we really coherently embark on an integrated malaria control that will eventually lead to malaria elimination/eradication.

## Abbreviations

ACT: Artemisinin-based combination therapies; AIDS: Acquired immune deficiency syndrome; CI: Confidence interval; CSRS: Centre Suisse de Recherches Scientifiques en Côte d’Ivoire; Global Fund: Global Fund to Fight AIDS, Tuberculosis and Malaria; HIV: Human immunodeficiency virus; OR: Odds ratio; RDT: Rapid diagnostic test.

## Competing interests

The authors declare that they have no competing interests.

## Authors’ contributions

CCC implemented the study and drafted the manuscript. AFO contributed to the study implementation, analysis and interpretation of the data and drafted and revised the manuscript. BGK designed the study and assisted in all steps of study implementation, data analysis and interpretation and revision of the manuscript. GR contributed to the design of the study and the revision of the manuscript. MT and JU contributed to the design of the study, interpretation of the results and the revision of the manuscript. All authors read and approved the final manuscript.

## Pre-publication history

The pre-publication history for this paper can be accessed here:

http://www.biomedcentral.com/1471-2458/12/1089/prepub
